# Comparing the Relationship Between Age and Length of Disability Across Common Chronic Conditions

**DOI:** 10.1097/JOM.0000000000000702

**Published:** 2016-05-09

**Authors:** Arif Jetha, Elyssa Besen, Peter M. Smith

**Affiliations:** Liberty Mutual Research Institute for Safety, Hopkinton, Massachusetts (Drs Jetha, Besen); Institute for Work & Health (Drs Jetha, Smith); Dalla Lana School of Public Health, University of Toronto, Ontario, Canada (Dr Smith); and School of Public Health and Preventive Medicine, Monash University, The Alfred Centre, Melbourne, Victoria, Australia (Dr Smith).

## Abstract

**Objective:**

The aim of this study was to compare the association between age and disability length across common chronic conditions.

**Methods:**

Analysis of 39,915 nonwork-related disability claims with a diagnosis of arthritis, diabetes, hypertension, coronary artery disease, depression, low back pain, chronic pulmonary disease, or cancer. Ordinary least squares regression models examined age-length of disability association across chronic conditions.

**Results:**

Arthritis (76.6 days), depression (63.2 days), and cancer (64.9 days) were associated with longest mean disability lengths; hypertension was related to shortest disability lengths (41.5 days). Across chronic conditions, older age was significantly associated with longer work disability. The age–length of disability association was most significant for chronic pulmonary disease and cancer. The relationship between age and length of work disability was linear among most chronic conditions.

**Conclusions:**

Work disability prevention strategies should consider both employee age and chronic condition diagnosis.

A graying labor force coupled with an increase in disease-related risk factors has meant that the number of adults working with a chronic health condition is expected to increase substantially over the next decade.^1,2^ Emerging studies show that both the prevalence of chronic conditions and the frequency and duration of sickness absence increases in older ages.^1–4^ These trends reflect significant challenges for policy and program planners who need to develop targeted work disability prevention strategies that can be applicable at a population level and address the growing proportion of older workers. Highlighting an important research gap, studies have not examined whether the association between age and length of disability varies across multiple commonly reported chronic conditions.

Work disability is a significant concern for the 133 million Americans living with one or more chronic condition, and contributes to over one trillion dollars in lost productivity costs/year.^1,2,4^ Studies spanning different chronic conditions describe complex interrelationships between health-related symptoms (eg, pain, fatigue, activity limitation), self-management requirements, barriers or facilitators in the workplace (eg, flexibility, availability of accommodation, and workplace social support), and employment participation restrictions.^5–7^ Accordingly, people living with a chronic condition often have work absences, covered by short-term disability (STD) or long-term disability (LTD) insurance, to manage their condition, or to regain the strength and energy that they require to perform their job. Several studies have compared different chronic conditions in terms of their impact on work disability. Although findings vary depending on the study sample, they tend to show that conditions, including depression, coronary artery disease, cancer, chronic pulmonary disease, and arthritis, can be associated with prolonged periods of sickness absence.^8–11^

The relationship between chronic disease and length of disability may also be associated with older age.^11^ Close to 80% of adults aged 50 years or older have at least one chronic condition.^2,12–14^ The high prevalence of chronic conditions at older ages coupled with age-related functional changes (eg, diminished physical capacity, slowing cognition, decreased working memory, and difficulty with hearing and vision) may contribute to longer sickness absences.^11,15–17^ A recent examination of 239,359 work disability claims found a 17-day difference in length of disability when comparing younger (25 years of age) to older adults (65 years of age).^18^ When controlling for job tenure, older age was significantly associated with a greater length of disability.^18^ The association between age and length of disability might not be consistent across chronic conditions and could vary based on a number of factors, including rate of functional decline and recovery time, personal motivation to return-to-work, availability of workplace supports, or disability coverage.^15,17,19–21^ At the same time, advancing age may provide benefits to employment. Older adults often have more work experiences, workplace attachment, and job control and autonomy than less experienced younger adults who may report more precarious employment.^15,17,22,23^ Older adults may also be better able to balance participation in work and nonwork roles than younger and middle-aged workers.^24^

Few studies have examined how the relationship between age and length of disability may vary across multiple chronic conditions. In a study of Canadian workers’ compensation claimants reporting a work-related musculoskeletal injury, the association among chronic disease comorbidity, length of sickness absence, and age was examined.^25^ Findings showed a significant indirect effect of older age and greater length of sickness absence for those experiencing a work-related musculoskeletal injury and who also had a diagnosis of diabetes, arthritis, or coronary arthritis disease.^25^ Study findings also showed that for people living with depression, length of sickness absence peaked in the middle age groups suggesting that differences in length of disability exist between people living with physical and mental health conditions at different ages.^25^ Similar comparative analyses examining the association between age and work disability length of nonworked-related claims resulting directly from chronic conditions have not been conducted. This study aims at examining how the relationship between age and length of disability varies across different chronic conditions. Findings have implications for public health professionals and policy makers designing strategies aimed at preventing work disability for people living with a broad range of chronic conditions.

## METHODS

Data for this study were drawn from administrative records of a large private disability insurance company in the US. All STD and LTD claims from 2008 through 2012 for nonwork-related conditions were considered for inclusion, and were followed for 1 year from the start of disability, or until the close of the claim if occurring before 1 year. In the US, STD and LTD coverage provides wage replacement for individuals needing to take time from work for health-related reasons.^26^ We focused on eight commonly reported health conditions that are chronic in nature, doctor-diagnosed, and indicated as the primary reason for work disability, including arthritis, cancer, chronic pulmonary disease, coronary artery disease, diabetes, depression, hypertension, and low back pain.^2,4,9,10^ ICD-9 codes, presented in Appendix Table 1, were selected on the basis of previously published research and used to select claims.^25,27^ Claims represented a variety of different employers, industries, states, and company sizes. This study was approved by the New England Institutional Review Board.

**FIGURE 1:**
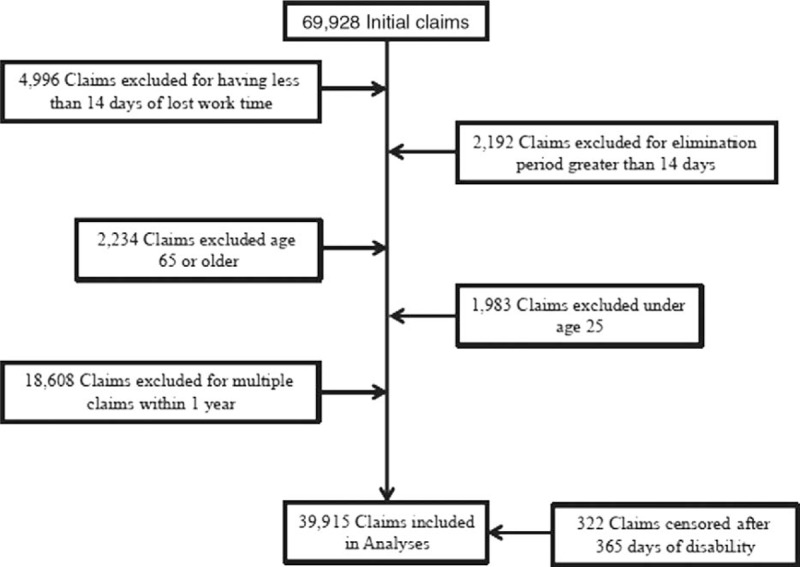
Descriptions of study exclusions for claimants included in the analyses.

Several exclusions were used in this study (Fig. 1). In STD and LTD cases, an average waiting period from the time in which an individual reported needing time off of work and commencement of wage replacement varied between 7 and 14 days, depending on company policy. Over 95% of the claims had a waiting period of 14 days or less. To account for these differences, our sample was restricted to those who had a minimum of 14 days of disability, which included the days in the waiting period, and a waiting period of l4 days or less. To ensure an adequate sample size and comparability across ages, claimants aged less than 25 years and over the age of 65 years were excluded. Individuals under the age of 25 represent those in a unique transitional life phase who are not in their primary career, and chronic condition-related disability claims are not highly prevalent. For those aged 65 years or older, Social Security is the primary form of disability wage replacement in the US. Accordingly, STD and LTD cases for adults aged 65 years or older in the database have restricted coverage and may not be comparable to claimants under the age of 65 years. Finally, we excluded those who filed multiple claims within a single calendar year due to challenges attributing work disability to a particular condition. Overall, there were 39,915 claims in the analyses.

## MEASURES

### Length of Disability

Length of disability was calculated from the start of disability to when wage replacement benefits ended. In claims wherein wage replacement benefits had not ended within 365 days of the start of disability, the claim was censored to have a value of 365. This occurred in 322 claims. The distribution for length of disability was highly skewed. Accordingly, the natural log of length of disability was used as the outcome variable in analyses.

### Chronic Health Condition

International Classification of Diseases Ninth Edition (ICD-9) codes for the primary cause of work disability were utilized to select cases included in the analysis, and were based on previously published research.^25^ We included claims with diagnoses for arthritis, diabetes, hypertension, coronary artery disease, depression, low back pain, chronic pulmonary disease, or cancer (see Table 1).

### Age

Age was measured in years on the basis of claimant's age when wage replacement first started.

### Covariate

Several covariates were used in analyses on the basis of evidence of their relationship with length of disability.^28,29^ Covariates included gender (1 = female; 0 = male), marital status (1 = married and 0 = unmarried), job tenure (years since date of hire), hours worked per week (part-time ≤34 hours/week, full-time 35 to 44 hours/week, and overtime ≥45 hours/week), annual income (coded as $10,000 increments for $0 to $149,999; annual income of ≥$150,000 was categorized as a single group), and physical job demands (1 = for sedentary, 2 = light demands, 3 = medium demands, 4 = heavy demands, and 5 = very heavy demands). Industry was categorized into eight groups to match the U.S. Department of Labor's Standard Industry Classification (SIC),^30^ including construction, finance and insurance (reference group), manufacturing, mining, retail trade, services, transportation, and wholesale trade. The agriculture, forestry and fishing, and public administration industries were not represented in the database. Across employers represented in the database, there were different time points at which claimants made the transition from STD to LTD based on the employers’ benefits plan. To control for these differences, a continuous variable indicating duration of STD benefits based on different employer benefit plans was included as a covariate in analyses.

### Analyses

Ordinary least squared (OLS) regression models were used to examine the relationship between age and length of disability across the eight chronic health conditions. In models, standard errors were clustered on the basis of the claimant's industry grouping. As claimants are nested within industries, it is likely that observations within the same industry group may not be truly independent as OLS assumes. Therefore, clustering the standard errors by industry was implemented to take nonindependence into account. In addition, the industry groupings were included as covariates in analyses to further adjust for nonindependence of observations within industry groups.

Separate models were estimated for each chronic health condition. The main predictor variable in the analyses was age. To account for possible nonlinearity in the age-length of disability relationship, both age and age squared terms were included in the models. All analyses were adjusted for tenure, gender, hours worked/week, income, marital status, physical job demands, length of STD benefits, and industry. Continuous variables in the models were mean-centered to reduce issues with multicollinearity. Analyses were also stratified by gender to determine whether findings differed when comparing males and females.

To compare coefficients across the different models for the chronic health conditions, the postestimation command seemingly unrelated estimate (SUEST) was used. SUEST allowed for the combination of estimation results across unrelated subsamples for the different chronic conditions. Wald tests were used to compare both the age coefficients and the predicted values for length of disability when all variables in the models were held at their means across the chronic health condition models. Analyses were performed using STATA 13.^31^

## RESULTS

There were 39,915 claims included in the analyses. The average length of tenure for the claimants was 7.2 years. Slightly less than half of the claimants were male (41.6%), a majority were full-time workers (92.6%), and about half were married (48.1%). Almost three-quarters of the claimants (73.1%) had an annual income of $50,000 or less. More than half of the sample (58.8%) worked in jobs that were sedentary or had light physical job demands and 13.0% worked in jobs with heavy or very heavy physical job demands. A summary of the sample characteristics is provided in Appendix Table 2.

As seen in Table 1, there were over 1000 claimants in each of the chronic condition subsamples with the exception of diabetes (916 claimants). The most prevalent chronic conditions in our sample were depression, low back pain, and cancer. There were differences across the chronic health conditions in the number of claims by age group. For depression, a greater percentage of claimants were in the younger age groups relative to the other health conditions. For arthritis, chronic pulmonary disease, and coronary artery disease, a greater percentage of claimants were in the older age groups. The average age of claimants was 45.3 years. However, the mean age of claimants significantly differed across chronic health conditions (F = 819.51, *P* < 0.001): arthritis (50.8 years), diabetes (46.8 years), hypertension (46.2 years), coronary artery disease (52.1 years), depression (41.1 years), low back pain (43.8 years), chronic pulmonary disease (50.2 years), and cancer (49.1 years).

**FIGURE 2:**
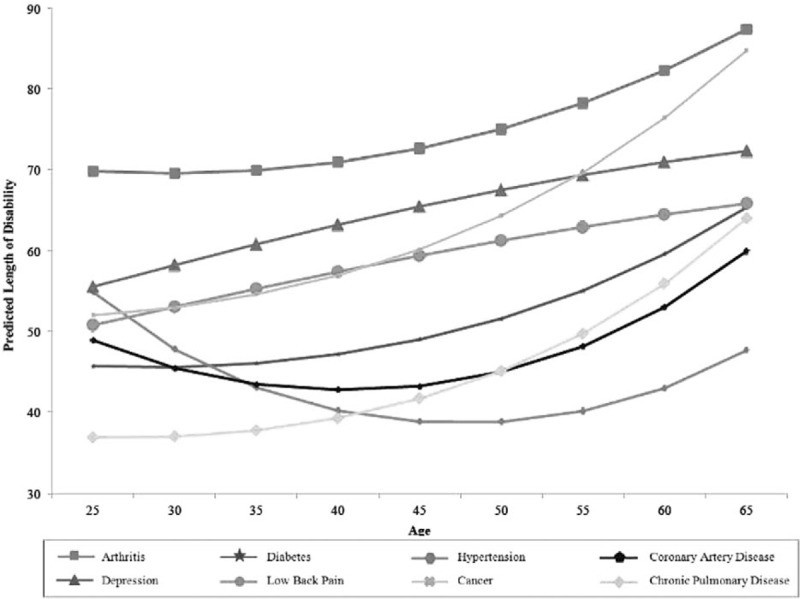
Relationship between predicted length of disability and age for chronic health conditions examined in this study.

For all chronic conditions, there was a significant relationship between age and length of disability (Table 2). As illustrated in Fig. 2, the relationship between older age and length of disability was linear and positive across all chronic conditions with the exception of cancer, hypertension, and chronic pulmonary disease, which exhibited a nonlinear relationship. For hypertension, the predicted length of disability decreased with age until 45 years, where the predicted length of disability began to significantly increase with age. For cancer, from ages 25 to 45, the predicted length of disability increased relatively little with age, while after 45 years, it increased at a sharper rate with older age. Similarly, chronic pulmonary disease–related length of disability grew at a sharper rate after 40 years. When compared with cancer and hypertension, nonlinearity in the relationship between age and length of disability was not significant for those living with chronic pulmonary disease. The relationship between age and length of disability did not tend to differ when comparing male and females living with most of the common chronic conditions examined.

Analyses of coefficients across the chronic condition models compared the mean predicted length of disability for each of the chronic conditions (Table 3). Arthritis was predicted to have a significantly greater length of disability at 76.6 days than all of the other conditions. Both depression (63.2 days) and cancer (64.9 days) also had relatively longer predicted disability durations than the other conditions with the exception of arthritis. Hypertension was predicted to have the shortest length of disability at 41.5 days, which was significantly shorter than all of the other conditions with the exception of chronic pulmonary disease (47.0 days).

The coefficients for age were also examined across the models (Table 3). The largest coefficients for the age-length of disability relationship were for chronic pulmonary disease and cancer. The predicted length of disability for cancer ranged from a low of 51.9 days (25 years of age), to 84.7 days (65 years). For chronic pulmonary disease, the predicted length of disability varied by 27.0 days, from 36.8 days (25 years) to 64.0 days (65 years). Hypertension, depression, and low back pain all had relatively weaker relationships varying by approximately 15 to 16 days across the age range. For hypertension, the predicted length of disability ranged from a high of 54.8 days at age 25, to a low of 38.7 days at age 50, and then back up to 47.6 days at age 65. The predicted length of disability ranged from 55.5 to 72.3 days for depression and 50.7 to 65.8 days for low back pain. Sensitivity analyses were conducted to assess whether the study findings varied if the claims having a length of disability of more than 365 days were excluded from the models. The results remained consistent when the 322 claims were removed, suggesting that they were not overly biasing the results. Sensitivity analyses are available upon request.

## DISCUSSION

This study was one of the first to compare age and length of work disability across common chronic conditions. Findings from the analysis of STD and LTD claims showed that while advancing age was associated with greater work disability length, the specific relationship between age and disability duration differed across conditions. Arthritis, depression, and cancer were associated with the longest disability. Results also exhibited an increasing linear relationship between age and length of disability for most conditions examined, with the exception of cancer and hypertension. Findings highlight the importance of work disability prevention initiatives at older ages, and underscore the need to pay special attention to chronic disease diagnosis to identify those who may be at a particular risk for prolonged sickness absences.

Within the context of an aging workforce, results from this study found that both the proportion and length of disability claims increased with age. Findings reflect the potential work-related impact of a chronic condition on older adults. A chronic condition may exacerbate physical and cognitive changes associated with aging, and contribute to longer disability duration when compared with younger and middle-aged adults.^11,15,18^ Results highlight the need to design work disability prevention strategies tailored to older adults, regardless of the health condition they report, to minimize work disability. Preventing work disability in older ages may have important long-term personal (eg, delayed labor market exit, financial security in later years, continued access to health insurance that is important to chronic condition management) and social implications (eg, minimizing the burden older workers may have on private workers compensation and public social security).

Not surprisingly, arthritis diagnosis was associated with the greatest mean length of disability claims. Previous research has shown that arthritis is the most significant cause of work disability in industrialized countries and is associated with loss of function, workplace participation restrictions, and time required for self-management.^7,13,14^ Interestingly, cancer and depression were also associated with greater mean length of disability than other chronic conditions. Although arthritis, cancer, and depression are clinically different, they could be characterized by similar health-related risk factors, including pain, fatigue, and activity limitations that could be associated with longer disability duration.^32–36^ These conditions could also be characterized as invisible (eg, no easily identifiable signs or symptoms) and/or episodic (eg, unpredictable disease activity), which may make early disclosure, availability of workplace social support, and accessibility of job accommodations challenging.^37^ Research is required to better understand the health characteristics and work experiences common to a range of chronic conditions to design work disability prevention strategies that are broadly applicable.

The relationship between age and length of disability tended to increase linearly for all conditions except cancer and hypertension, which exhibited a significant nonlinear association. Along with the functional changes associated with aging with a chronic condition, findings could be explained using a life course perspective that suggests that a person's age is related to socially defined milestones that shape involvement in work.^24^ Through a life course perspective, older adulthood could be characterized as a time of life where a person is withdrawing or rearranging participation in roles such as employment and may have less motivation to return-to-work from a sickness absence. In comparison, young and middle-aged adults may have more concerns about prolonged work disability on their ability to support involvement in a greater range of personal and work roles, and take more steps to minimize sickness absence. Interestingly, the age-length of disability relationship was strongest for cancer and chronic pulmonary disease with length of disability increasing exponentially in the middle years. It may be that treatment of these conditions might accelerate biological aging and result in increases in disability duration.^38,39^ It may also be that comorbidities associated with both chronic conditions significantly influence the ability to return-to-work following sickness absence. For claimants reporting hypertension, length of disability exhibited a U-shape relationship with age. For young and older adults, hypertension may have severe underlying causes (eg, kidney, endocrine, or heart disease) that are challenging to manage.^40,41^ Variability in hypertension screening and challenges with self-management could also contribute to work disability severity at these ages.^40^ Findings highlight the complex relationship between age and length of disability among people with different chronic conditions and the need for additional research to understand the mechanisms that underlie the association. Although gender did not attenuate the relationship between age and length of disability across most of the chronic conditions examined, we suggest further gender-based comparisons to better understand the association between chronic disease and work disability.

Study strengths include the large and diverse sample of workers representing a range of geographic regions, industries and chronic conditions, and objective outcome data on length of disability. Study limitations should be recognized. Findings were based on a cross-sectional design that was unable to ascertain causality, as well as generational differences that could account for variations in length of disability. Information captured in the administrative database was also a limitation. Details on condition severity (eg, levels of pain, fatigue, and disease activity), previous chronic disease diagnosis, presence of comorbidities, and work context characteristics (eg, company size, social support, job demands, or availability of accommodations) were unavailable. Although this information was unavailable in our database, these factors might be sources of variation that should be examined in future research. In this study, we focused on the time until the end of disability benefits as a marker of return-to-work. However, there is no information about whether a claimant actually returned to work when he or she stopped receiving disability benefits. Accordingly, findings may have captured claimants that were listed as relatively short in duration but were actually permanently disabled. Lastly, the scope of STD and LTD policies in the US may have restricted study findings. In comparison to STD, which is often provided by US employers, LTD benefits are voluntary. Although most claims in our analysis did not approach LTD, there is a possibility that the length of disability for some claims might have reflected the end of STD benefit rather than return-to-work. At the longest disability durations, our sample may have been biased to those individuals who opted to have LTD benefits.^18^

The growing proportion of Americans who are working in their later years and living with a chronic condition underscores the need for work disability research that spans the life course. This study was one of the first to compare how different chronic health conditions are related to work disability length at different ages. Findings indicated that increasing age was associated with a greater length of disability among all conditions that were examined. Results also point to arthritis, cancer, and depression being related to relatively longer work disability. Although the age-length of disability relationship increased linearly for most chronic conditions, cancer and hypertension exhibited a significant nonlinear relationship. The nexus between age and the type of health condition that an individual is living with can play an important role in their work experiences and should be considered in future research and population health interventions aimed preventing work disability.

## Supplementary Material

Supplemental Digital Content

## Supplementary Material

Supplemental Digital Content

## Figures and Tables

**TABLE 1:** Number of Claims Within Each Chronic Health Condition by Age Groups

Chronic Health Condition	Claims by Age Group								
	25–29	30–34	35–39	40–44	45–49	50–54	55–59	60–64	Total
	*n* (%)	*n* (%)	*n* (%)	*n* (%)	*n* (%)	*n* (%)	*n* (%)	*n* (%)	*n*
Arthritis	40 (3.3%)	46 (3.8%)	88 (7.3%)	126 (10.5%)	194 (16.1%)	251 (20.8%)	242 (20.1%)	217 (18.0%)	1,204
Diabetes	46 (5.0%)	84 (9.2%)	104 (11.4%)	165 (18.0%)	151 (16.5%)	153 (16.7%)	127 (13.9%)	86 (9.4%)	916
Hypertension	68 (6.4%)	113 (10.6%)	132 (12.3%)	163 (15.2%)	190 (17.8%)	149 (13.9%)	142 (13.3%)	112 (10.5%)	1,069
Coronary artery disease	11 (0.5%)	59 (2.6%)	108 (4.7%)	250 (10.9%)	419 (18.2%)	524 (22.8%)	529 (23.0%)	402 (17.5%)	2,302
Depression	1809 (14.8%)	2217 (18.2%)	2025 (16.6%)	1817 (14.9%)	1670 (13.7%)	1364 (11.2%)	884 (7.3%)	407 (3.3%)	12,193
Low back pain	1197 (10.8%)	1512 (13.7%)	1628 (14.7%)	1643 (14.9%)	1702 (15.4%)	1479 (13.4%)	1134 (10.3%)	768 (6.9%)	11,063
Chronic pulmonary disease	50 (4.2%)	65 (5.5%)	106 (9.0%)	113 (9.6%)	168 (14.3%)	223 (18.9%)	237 (20.1%)	216 (18.3%)	1,178
Cancer	372 (3.7%)	596 (6.0%)	907 (9.1%)	1396 (14.0%)	1707 (17.1%)	1857 (18.6%)	1751 (17.5%)	1404 (14.1%)	9,990

*n* = number of claims. % = percentage of claims within each age group by chronic health condition.

**TABLE 2:** Ordinary Least Squared Regression Model Results for Age Predicting the Length of Disability by Chronic Health Conditions

	Arthritis	Diabetes	Hypertension	Coronary Artery Disease	Depression	Low Back Pain	Chronic Pulmonary Disease	Cancer
Predictors	b (SE)	b (SE)	b (SE)	b (SE)	b (SE)	b (SE)	b (SE)	b (SE)
Age^a^	0.077 (0.030)^**^	0.099 (0.007)^***^	−0.019 (0.020)	0.131 (0.035)^***^	0.072 (0.005)^***^	0.067 (0.013)^***^	0.178 (0.032)^***^	0.142 (0.013)^***^
Age^2^	0.018 (0.025)	0.027 (0.018)	0.069 (0.017)^***^	0.056 (0.029)	−0.008 (0.009)	−0.007 (0.007)	0.038 (0.023)	0.025 (0.009)^**^
Female ^b^	0.012 (0.034)	−0.025 (0.046)	−0.016 (0.039)	0.016 (0.081)	0.071 (0.014)^***^	0.037 (0.015)^*^	−0.125 (0.049)^*^	−0.012 (0.032)
Married^c^	0.036 (0.029)	0.029 (0.069)	−0.033 (0.065)	−0.037 (0.032)	0.016 (0.007)^*^	0.030 (0.015)^*^	−0.034 (0.035)	0.005 (0.013)
Income^d^	−0.032 (0.004)^***^	0.013 (0.009)	0.027 (0.012)^*^	−0.010 (0.009)	0.019 (0.005)^***^	−0.008 (0.003)^**^	−0.017 (0.018)	−0.009 (0.005)
Job tenure^e^	0.056 (0.015)^***^	−0.008 (0.022)	−0.026 (0.025)	0.001 (0.016)	−0.009 (0.005)	−0.015 (0.009)	−0.008 (0.018)	0.005 (0.009)
Full-time^f^	−0.003 (0.050)	0.322 (0.121)^**^	0.119 (0.103)	0.145 (0.112)	0.119 (0.038)^**^	0.096 (0.017)^***^	0.376 (0.153)^*^	0.193 (0.044)^***^
Overtime	0.080 (0.078)	0.010 (0.125)	0.136 (0.068)^*^	0.040 (0.041)	−0.089 (0.052)	−0.025 (0.034)	0.096 (0.173)	−0.033 (0.042)
Physical job demands	0.055 (0.034)	0.028 (0.070)	0.032 (0.023)	0.072 (0.022)^***^	−0.049 (0.014)^***^	0.028 (0.019)	0.062 (0.037)	−0.002 (0.019)
Industry^g^								
Construction	0.728 (0.049)^***^	−0.640 (0.062)^***^	0.002 (0.031)	0.032 (.067)	−0.139 (0.075)	0.072 (0.046)	0.271 (0.073)^***^	−0.152 (0.034)^***^
Manufacturing	−0.089 (0.052)	−0.053 (0.091)	−0.176 (0.021)^***^	−0.057 (0.021)^**^	−0.094 (0.008)^***^	−0.123 (0.021)^***^	0.347 (0.059)^***^	−0.072 (0.013)^***^
Mining	−0.216 (0.077)^**^	1.046 (0.176)^***^	0.052 (0.063)	−0.177 (0.037)^***^	0.165 (0.022)^***^	−0.388 (0.017)^***^	0.495 (0.110)^***^	0.046 (0.015)^**^
Retail	0.007 (0.054)	0.055 (0.118)	0.150 (0.044)^***^	0.024 (0.063)	−0.011 (0.059)	0.145 (0.042)^***^	0.241 (0.074)^***^	0.025 (0.040)
Services	−0.031 (0.049)	0.139 (0.074)	−0.003 (0.020)	−0.016 (0.023)	−0.004 (0.041)	0.104 (0.029)^***^	0.087 (0.038)^*^	−0.020 (0.016)
Transportation	−0.081 (0.030)^**^	−0.176 (0.052)^***^	−0.090 (0.027)^***^	−0.103 (0.033)^**^	0.038 (0.010)^***^	−0.081 (0.020)^***^	0.051 (0.032)	−0.092 (0.012)^***^
Wholesale	0.056 (0.047)	−0.165 (0.085)	−0.183 (0.036)^***^	0.030 (0.045)	0.035 (0.035)	−0.006 (0.032)	0.182 (0.072)^*^	0.041 (0.021)
STD Max Days	0.002 (0.000)^***^	0.001 (0.001)	0.002 (0.001)^***^	0.001 (0.001)^*^	0.002 (0.001)	0.002 (0.001)^***^	0.001 (0.000)^***^	0.002 (0.001)^***^
Intercept^h^	4.318 (0.045)^***^	3.908 (0.070)^***^	3.687 (0.052)^***^	3.869 (0.062)^***^	4.102 (0.017)^***^	4.030 (0.012)^***^	3.746 (0.072)^***^	4.171 (0.028)^***^

STD, short-term disability days.

^a^Age coded in 10 s of years (eg, one unit of age is 10 yrs).

^b^Male is the reference group.

^c^Unmarried is the reference group.

^d^Income coded in 16 groups with $10,000 increments for 1 = $0–10,000 = 1; 16 = ≥$150,000.

^e^Tenure coded in 5 s of years (eg, one unit of tenure is 5 yrs).

^f^Part-time is the reference group.

^g^Finance and insurance is the reference group.

^h^Natural log of length of disability.

^*^P < 0.05.

^**^P < 0.01.

^***^P < 0.001.

**TABLE 3:** Chronic Health Condition Model Comparisons

Chronic Health Condition	Mean Predicted LOD^*^	Pairwise Comparisons of LOD ^†^	Pairwise Comparisons of Age Coefficients^†^
1. Arthritis	76.640	1>2, 3, 4, 5, 6, 7, 8	1<7
2. Diabetes	51.061	2<1, 5, 8; 2>3, 7	2>5, 6
3. Hypertension	41.541	3<1, 2, 4, 5, 6, 8	3<4, 7, 8
4. Coronary artery disease	47.724	4<1, 5, 6, 8; 4>3	4>3, 5, 6
5. Depression	63.196	5<1; 5>2, 3, 4, 6, 7	5<2, 4, 7, 8
6. Low back pain	58.454	6<1, 5, 8; 6>3, 4, 7	6<2, 4, 7, 8
7. Chronic pulmonary disease	46.951	7<1, 2, 5, 6, 8	7>1, 3, 5, 6
8. Cancer	64.909	8<1; 8>2, 3, 4, 6, 7	8>3, 5, 6

^*^Represents the mean predicted length of disability based on the regression model with all variables in the model held at their mean.

^†^Comparisons based on Wald tests with *P* < 0.05.
