# Supplementary material for: Comparing the Relationship Between Age and Length of Disability Across Common Chronic Conditions
Source: J Occup Environ Med. 2016 May 9;58(5):485–91. doi: 10.1097/JOM.0000000000000702 (PMC4857792; doi:10.1097/JOM.0000000000000702)
Supplement: Supplemental Digital Content [file joem-58-485-s001.docx]

Appendix Table 2: Summary of ICD-9 codes for commonly reported chronic conditions utilized in the analyses.

| **Chronic Health Condition** | **ICD-9 Codes** |
| --- | --- |
|  |  |
|  |  |
| Arthritis | 714-715 |
| Diabetes | 250 |
| Hypertension | 401-405 |
| Coronary Artery Disease | 410-414 |
| Depression | 311; 300.4; 296.2; 296.3 |
| Low Back Pain | 344.6; 353.1; 353.4; 353.8; 353.9; 355.0; 721.42; 722.1; 722.73; 722.83; 724.02; 724.3; 724.4; 724.6; 738.4; 952.2; 952.3; 952.4; 952.8; 953; 956.0; 720.2; 721.3; 722.32; 722.5; 722.93; 724.2; 724.5; 724.7; 737.3; 737.4; 739.3; 739.4; 839.2; 939.41; 839.42; 846; 847.2; 847.3; 847.4 |
| Chronic Pulmonary Disease | 491-494; 496; 506.4 |
| Cancer | 140-239 |
