# Supplementary material for: Comparing the Relationship Between Age and Length of Disability Across Common Chronic Conditions
Source: J Occup Environ Med. 2016 May 9;58(5):485–91. doi: 10.1097/JOM.0000000000000702 (PMC4857792; doi:10.1097/JOM.0000000000000702)
Supplement: Supplemental Digital Content [file joem-58-485-s002.docx]

| Table 1: Number of claims within each chronic health condition by age groups. | | | | | | | | | | | | | | | | |  |  |  |
| --- | --- | --- | --- | --- | --- | --- | --- | --- | --- | --- | --- | --- | --- | --- | --- | --- | --- | --- | --- |
| Chronic Health Condition | Claims by Age Group | | | | | | | | | | | | | | | | |  |  |
|  | 25-29 | 30-34 | | 35-39 | | 40-44 | | 45-49 | | 50-54 | | 55-59 | | 60-64 | | Total | |  |  |
|  | n(%) | n(%) | | n(%) | | n(%) | | n(%) | | n(%) | | n(%) | | n(%) | | n | |  |  |
| Arthritis | 40(3.3%) | 46(3.8%) | | 88(7.3%) | | 126(10.5%) | | 194(16.1%) | | 251(20.8%) | | 242(20.1%) | | 217(18.0%) | | 1204 | |  |  |
| Diabetes | 46(5.0%) | 84(9.2%) | | 104(11.4%) | | 165(18.0%) | | 151(16.5%) | | 153(16.7%) | | 127(13.9%) | | 86(9.4%) | | 916 | |  |  |
| Hypertension | 68(6.4%) | 113(10.6%) | | 132(12.3%) | | 163(15.2%) | | 190(17.8%) | | 149(13.9%) | | 142(13.3%) | | 112(10.5%) | | 1069 | |  |  |
| Coronary Artery Disease | 11(0.5%) | 59(2.6%) | | 108(4.7%) | | 250(10.9%) | | 419(18.2%) | | 524(22.8%) | | 529(23.0%) | | 402(17.5%) | | 2302 | |  |  |
| Depression | 1809(14.8%) | 2217(18.2%) | | 2025(16.6%) | | 1817(14.9%) | | 1670(13.7%) | | 1364(11.2%) | | 884(7.3%) | | 407(3.3%) | | 12193 | |  |  |
| Low Back Pain | 1197(10.8%) | 1512(13.7%) | | 1628(14.7%) | | 1643(14.9%) | | 1702(15.4%) | | 1479(13.4%) | | 1134(10.3%) | | 768(6.9%) | | 11063 | |  |  |
| Chronic Pulmonary Disease | 50(4.2%) | 65(5.5%) | | 106(9.0%) | | 113(9.6%) | | 168(14.3%) | | 223(18.9%) | | 237(20.1%) | | 216(18.3%) | | 1178 | |  |  |
| Cancer | 372(3.7%) | 596(6.0%) | | 907(9.1%) | | 1396(14.0%) | | 1707(17.1%) | | 1857(18.6%) | | 1751(17.5%) | | 1404(14.1%) | | 9990 | |  |  |
| **Notes:**  n=number of claims | | |  | |  | |  | |  | |  | |  | |  | | |  |  |
| %=percentage of claims within each age group by chronic health condition | | | | | | | | | | | | | | | | |  |  |  |
